# Kooperation zwischen dem Health Data Hub und dem FDZ Gesundheit am BfArM beim Aufbau des europäischen Gesundheitsdatenraums

**DOI:** 10.1007/s00103-023-03829-7

**Published:** 2024-01-10

**Authors:** Roxana Arzideh, Mario Jendrossek, Irene Zittlau

**Affiliations:** Forschungsdatenzentrum Gesundheit, Bundesinstitut für Arzneimittel und Medizinprodukte (BfArM), Dienstsitz Bonn, Kurt-Georg-Kiesinger Allee 3, 53175 Bonn, Deutschland

**Keywords:** EHDS, Interoperabilität, Gesundheitsdaten, Digitalisierung, Sekundäre Gesundheitsdaten, EHDS, Interoperability, Health Data, Digitalization, Secondary health data

## Abstract

In den vergangenen Jahren hat der grenzübergreifende Austausch der europäischen Mitgliedstaaten in Bezug auf die Nutzung von Gesundheitsdaten an Relevanz zugenommen. Um der aktuell bestehenden Heterogenität und den gesundheitsbezogenen Herausforderungen gemeinschaftlich entgegenzuwirken sowie die Vision des europäischen Gesundheitsdatenraums (EHDS) unter Einhaltung der hohen Datenschutzstandards sukzessive umzusetzen, wurden unterschiedliche Projekte mit Beteiligung des Health Data Lab (HDL) am Bundesinstitut für Arzneimittel und Medizinprodukte (BfArM) und des Health Data Hub (HDH) initiiert. Durch die enge deutsch-französische Kooperation werden einerseits Synergien geschaffen und Herausforderungen sowie Lösungsansätze europäisch beleuchtet. Andererseits fördert die Zusammenarbeit zwischen beiden Institutionen den grenzübergreifenden Wissens- und Erfahrungsaustausch, um auf nationaler und europäischer Ebene gemeinsam Herausforderungen zu begegnen und Ziele zu erreichen.

## Abstract (en)

In recent years, the cross-border exchange between European member states regarding the use of health data has become increasingly relevant. In order to jointly counteract the current heterogeneity and health-related challenges as well as to successively implement the vision of the European Health Data Space (EHDS) in compliance with high data protection standards, different projects with the participation of the Health Data Lab at the Federal Institute for Drugs and Medical Devices (BfArM) and the Health Data Hub have been initiated. This close German–French cooperation on the one hand creates synergies and examines challenges and possible solutions on a European level. On the other hand, the cooperation between both institutions promotes the cross-border exchange of knowledge and experience in order to jointly meet challenges and achieve goals at national and European levels.

## Hintergrund

Die COVID-19-Pandemie hat sowohl die Relevanz als auch die komplexen Hindernisse der Nutzung von Gesundheitsdaten deutlich aufgezeigt. Grenzübergreifende Kooperationen und der Austausch der europäischen Mitgliedstaaten in Bezug auf die Nutzung von Gesundheitsdaten bieten erhebliche Vorteile, um die medizinische Versorgung sowie die Prävention auf europäischer Ebene zu verbessern und die Entwicklung von wirksamen Therapien und Arzneimitteln sowie deren Einsatz voranzutreiben. Die Nutzung großer Datenmengen für weitere Zwecke weist ein enormes Potenzial auf, um evidenzbasierte, wissenschaftlich gesicherte medizinische Entscheidungen zu treffen. Über die COVID-19-Pandemie hinaus können Sekundärdaten im Gesundheitswesen grenzübergreifend einen Gewinn für die medizinische Versorgung der Bevölkerung Europas bringen und die Versorgungspraxis der Patientinnen und Patienten optimieren [1].

Um die Potenziale und Chancen der Digitalisierung für Europa zu nutzen, hat die Europäische Kommission für die aktuelle Legislaturperiode (2019–2024) den Schwerpunkt auf Digitalisierung gesetzt und das kommende Jahrzehnt zur *Digitalen Dekade Europas* erklärt [2]. Mit der Schaffung eines Binnenmarktes für Daten soll die globale Wettbewerbsfähigkeit und Datensouveränität Europas gewährleistet werden. Die Digitalisierungsstrategie beinhaltet insbesondere die Schaffung neun gemeinsamer europäischer Datenräume in verschiedenen Sektoren, wie beispielsweise der Landwirtschaft, Mobilität, öffentlichen Verwaltung und auch der Gesundheit [3]. Der europäische Gesundheitsdatenraum (European Health Data Space, EHDS) ist der erste sektorspezifische europäische Datenraum und ein Eckpfeiler der Europäischen Gesundheitsunion, die als Reaktion auf die COVID-19-Pandemie das Ziel verfolgt, den nationalen und europäischen Gesundheitsschutz zu verbessern und widerstandsfähigere europäische Gesundheitssysteme zu schaffen. Der zukünftige EHDS soll als gesundheitsspezifisches Ökosystem einen kohärenten, vertrauenswürdigen und effizienten Rahmen für die Nutzung von Gesundheitsdaten bieten und schließt sowohl die Ebene der Primärdatennutzung als auch die der Sekundärdatennutzung ein. Die Handlungskompetenz der Bürgerinnen und Bürger wird durch den unmittelbaren, kostenlosen und vereinfachten Zugang zu den eigenen elektronischen Gesundheitsdaten gestärkt und vorangetrieben durch die Einführung grenzüberschreitender (digitaler) Dienste in einem europaweit gemeinsamen technischen und semantischen Format. Zu den Diensten gehören sowohl elektronische Verschreibungen und Verabreichungen als auch Patientenkurzakten. Der Aufbau einer grenzüberschreitenden Infrastruktur der Primärdatennutzung, genannt MyHealth@EU, fördert somit eine effizientere Gesundheitsversorgung und höhere Patientensicherheit [1, 4].

Die Nutzung von sekundären Gesundheitsdaten bietet politischen Entscheidungsträgern, Regulierungsbehörden und Forschenden enorme Möglichkeiten einer verbesserten Gesundheitspolitik. Gewonnene Erkenntnisse fördern die evidenzbasierte Medizin und kommen der Gesundheitsversorgung der Bevölkerung zugute. Beispielsweise bedarf die Erforschung seltener Erkrankungen der Nutzung einer größtmöglichen Menge qualitativ hochwertiger Daten, um die Datengrundlage und folglich die Therapie zu verbessern. Um diese Daten verwenden zu können, wird im Rahmen des EHDS die sogenannte HealthData@EU-Infrastruktur aufgebaut. Gegenwärtig erschweren verschiedene Rechtsakte innerhalb der Mitgliedstaaten der Europäischen Union (EU) die grenzübergreifende Nutzung von Primär- und Sekundärdaten. Aufgrund dieser Heterogenität stellte die Europäische Kommission am 3. Mai 2022 einen Verordnungsentwurf zur Errichtung des EHDS vor (EHDS-VO). Der Entwurf baut auf die Datenschutzgrundverordnung auf und komplementiert zudem weitere europäische Gesetze und Verordnungen, u. a. aus den Bereichen Datenverwaltung (Governance), künstliche Intelligenz und Patientenrecht [5].

### Das Forschungsdatenzentrum Gesundheit in Deutschland

Das Forschungsdatenzentrum (FDZ) Gesundheit, auf internationaler Ebene „Health Data Lab (HDL)“, am Bundesinstitut für Arzneimittel und Medizinprodukte (BfArM) wurde im Jahr 2019 im Rahmen des Digitale-Versorgung-Gesetzes initiiert. Das FDZ Gesundheit befindet sich im Aufbau, der phasenweise erfolgen wird. Durch die Datentransparenzverordung (DaTraV) werden die gesetzlichen Regelungen konkretisiert [6]. Ziel des FDZ Gesundheit ist das Verfügbarmachen von Abrechnungsdaten aller gesetzlich Krankenversicherten des Spitzenverbandes (Bund der Krankenkassen, GKV-SV). Berechtigten Forschenden wird zukünftig in einer geschützten Analyseumgebung die systematische Forschung anhand dokumentierter und abgerechneter Gesundheitsleistungen aus der ambulanten und stationären Versorgung in Deutschland ermöglicht. Künftig wird der Datenbestand auch um Daten aus dem Bereich der sonstigen Leistungserbringer sowie der elektronischen Patientenakte (ePa) gemäß § 363 Fünftes Buch Sozialgesetzbuch (SGB V) erweitert. Somit werden Forschenden mit einer Nutzungsberechtigung gemäß §§ 303a bis 303f SGB V durch ein öffentliches Antragsportal in einer nutzerfreundlichen und datenschutzkonformen Umgebung aktuelle, vielfältige und umfangreiche Gesundheitsdaten für Analysezwecke zur Verfügung gestellt. Die Datenschutz- und IT-Sicherheitsmaßnahmen werden in enger Kollaboration mit dem Bundesbeauftragten für den Datenschutz und die Informationsfreiheit (BfdI) sowie dem Bundesamt für Sicherheit in der Informationstechnik (BSI) erarbeitet [6, 7].

Das FDZ Gesundheit ist des Weiteren auf europäischer Ebene in verschiedenen Projekten involviert.

### Der Health Data Hub in Frankreich

Der Health Data Hub (HDH) wurde im Jahr 2019 unter der französischen Rechtsform einer öffentlichen Interessenvereinigung („groupement d’intérêt public“) gegründet. Das Ziel ist es, einen einfachen und einheitlichen, transparenten sowie sicheren Zugang zu Gesundheitsdaten zu gewährleisten, um Forschung und Entwicklung zu unterstützen.

Der HDH ist gesetzlich damit beauftragt, die Daten des nationalen Gesundheitsdatensystems zu sammeln, zu organisieren und zur Verfügung zu stellen sowie Innovationen bei der Nutzung von Gesundheitsdaten zu fördern. Konkret erfüllt der HDH vier Hauptaufgaben:

1. Eine zentrale Anlaufstelle, die Projektträgerinnen und Projektträger bei administrativen Schritten wie beispielsweise dem Antragsprozess unterstützt.
2. Die Bereitstellung einer sicheren, dem neuesten Stand der Technik entsprechenden Plattform, die fortschrittliche Möglichkeiten zur Speicherung, Berechnung, Verknüpfung und Analyse von Daten bietet.
3. Die Bereitstellung eines Datenkatalogs mit Datensätzen von Partnerorganisationen. Dieser Katalog wird schrittweise und iterativ aufgebaut und ermöglicht durch die Replikation der Datenbanken den Zugang sowie die Verknüpfung mit weiteren Datenbanken.
4. Die Beschleunigung des Gesundheitsdaten-Ökosystems von Innovationen durch die Förderung des Erfahrungs- und Wissensaustauschs.

Die französischen Gesundheitsdaten sind für alle Projektträgerinnen und Projektträger, die zum öffentlichen Interesse beitragen, unter Einhaltung der gesetzlichen Vorschriften zugänglich. Private Akteurinnen und Akteure sind davon nicht ausgeschlossen. Dabei muss allerdings sichergestellt werden, dass der beantragte Datenzugang ebenfalls von öffentlichem Interesse ist.

Auf Grundlage der oben genannten französischen Rechtsreform, auf die der HDH basiert, wurde das Systéme National des Données de Santé (SNDS) um alle Daten erweitert, die mit einer Erstattung durch die Krankenversicherung verbunden sind. Das SNDS umfasst über medizinisch-administrative Daten hinaus zusätzlich Daten aus Registern, Forschungskohorten sowie Datensätze aus Krankenhäusern.

Damit die Nutzung von Gesundheitsdaten das volle Potenzial entfalten kann, muss die Bereitstellung der Daten weiter beschleunigt werden. In Frankreich genehmigt die Datenschutzbehörde Commission Nationale de l’Informatique et des Libertés (CNIL) die Verarbeitung von Gesundheitsdaten nach festgelegten Verfahren. Die Frist für die Beantragung der CNIL-Genehmigung, die in der Regel zwei Monate beträgt und einmal verlängert werden kann, führt aber in der Praxis häufig zu längeren Wartezeiten für die Antragstellenden. Darüber hinaus können auch die Vorbereitungen und der Abschluss von Verträgen mit den Dateninhabern langwierig sein. Daher wenden sich Akteurinnen und Akteure in einigen Fällen anderen, leichter zugänglichen Datenquellen im Ausland zu. Neben der Wahrnehmung der oben beschriebenen Aufgaben in Zusammenarbeit mit mehr als 100 Partnerorganisationen beteiligt sich der HDH ebenfalls an internationalen und europäischen Projekten und Initiativen [8].

### Synergien zwischen HDL am BfArM und HDH

Demzufolge ergeben sich deutliche inhaltliche Überschneidungen in den Aufgaben und Zielen des HDL am BfArM mit dem HDH sowie eine gemeinsame Vision des EHDS. Parallel zur Schaffung einer gesetzlichen Grundlage auf europäischer Ebene wurden verschiedene Fördermöglichkeiten geschaffen, die sowohl das HDL am BfArM als auch das HDH wahrgenommen haben. Die gemeinsamen Ziele und die Vision des EHDS haben zu einer grenzübergreifenden Zusammenarbeit zwischen HDL und HDH in unterschiedlichen Projekten geführt. Die deutsch-französische Kooperation setzt sich maßgeblich für die grenzübergreifende (sekundäre) Nutzung von Gesundheitsdaten ein. Durch die enge Zusammenarbeit werden Synergien geschaffen und Herausforderungen sowie Lösungsansätze europäisch beleuchtet. Die Zusammenarbeit fördert zudem den grenzübergreifenden Wissens- und Erfahrungsaustausch und treibt Innovationen voran, um auf nationaler und europäischer Ebene gemeinsam Herausforderungen zu begegnen und Ziele zu erreichen.

### Projekte

Insgesamt ergibt sich durch die enge Zusammenarbeit in verschiedenen Projekten (z. B. Joint Action Towards the European Health Data Space, TEHDAS [9] und HealthData@EU-Pilotprojekt [10]) über den grenzübergreifenden Austausch hinaus ein enormes Potenzial. TEHDAS hat das Ziel, die konzeptionelle Grundlage für den EHDS zu erarbeiten und voranzubringen, und wurde durch die EU-Kommission für die Laufzeit von Februar 2021 bis August 2023 kofinanziert. Die Koordination wurde durch den Finnish Innovation Fund Sitra übernommen; Institutionen aus 25 europäischen Ländern waren involviert. In insgesamt acht Arbeitspaketen wurden insbesondere die Schwerpunkte Teilnahme der Bürgerinnen und Bürger, Datenqualität/Interoperabilität, IT-Infrastruktur und Governance berücksichtigt [9]. Seit Kurzem ist TEHDAS abgeschlossen und die Konzepte dienen als Grundlage für die folgenden EHDS-Projekte.

Das HealthData@EU-Pilotprojekt wird ebenfalls durch die EU-Kommission kofinanziert und läuft bis Oktober 2024. Die Koordination des aus 17 europäischen Partnern bestehenden Konsortiums liegt beim HDH. Es besteht ein hoher Bedarf an einem experimentellen Aufbau des EHDS, da bis dato keine gemeinsame Infrastruktur existiert, die grenzübergreifend verschiedenen Institutionen den Zugang zu Gesundheitsdaten ermöglicht. Das Ziel des Pilotprojekts ist somit der Aufbau einer ersten Version des europäischen Gesundheitsdatenraums für die Sekundärnutzung von Gesundheitsdaten [10].

Das Projekt verbindet Datenplattformen in einer grenzübergreifenden Netzwerkinfrastruktur und entwickelt Dienste, welche die „User Journey“ (Nutzererfahrung) für Gesundheitsdaten-Forschungsprojekte auf EU-Ebene unterstützen. Zu den vorrangigen Diensten gehören Metadaten-Discovery sowie ein gemeinsames Formular zur Beantragung von Datengenehmigungen. Das Konsortium arbeitet eng mit der EU-Kommission zusammen, welche parallel die Entwicklung der sogenannten „zentralen Dienste“ (Central Services) für HealthData@EU vorantreibt. Um die Machbarkeit und das Potenzial der Wiederverwendung von Daten aus mehreren europäischen Ländern zu veranschaulichen, werden im Rahmen des Projekts fünf Anwendungsfälle durchgeführt, die ein breites Spektrum von Forschungsthemen umfassen und Daten aus mehreren europäischen Ländern nutzen [5, 10].

Über die im Folgenden näher beleuchteten zwei Projekte hinaus finden auch weitere gemeinsame Projektbeteiligungen statt: Zum Beispiel werden der HDH und das FDZ Gesundheit jeweils auf nationaler Ebene im Rahmen von Projekten, die im EHDS-VO vorgesehene zentrale Datenzugangs- und Koordinierungsstelle für die Nutzung von Gesundheitsdaten (auf internationaler Ebene sogenannte Health Data Access Bodies, HDABs) konzeptionieren, pilotieren und weiterentwickeln [11, 12]. Die EU-Kommission fördert diese Projekte in den jeweiligen Mitgliedstaaten, wodurch eine weitere Kooperationsschnittstelle zwischen dem HDH und dem FDZ Gesundheit vorliegt. Des Weiteren sind beide Institutionen innerhalb des Data Analysis & Real World Interrogation Network (DARWIN@EU) vernetzt. Das Projekt etabliert momentan eine zukunftsfähige Plattform für EU-Gesundheitsdaten der European Medicines Agency mit dem Ziel, sich zukünftig ebenfalls an den EHDS anzuschließen [13].

Im folgenden Abschnitt wird die Kollaboration zwischen dem französischen HDH und dem FDZ Gesundheit innerhalb der verschiedenen Projekte anhand der User Journey dargelegt.

#### Auffindbarkeit von Datensätzen

„Data Discovery“ ist der erste Schritt in der EHDS User Journey, also dem Weg von der Projektplanung über die Datennutzung bis zur Ergebnisveröffentlichung. Er soll prospektiven Datennutzenden ermöglichen, Datensätze zu identifizieren, die zu der wissenschaftlichen Fragestellung passen. Die EHDS-Verordnung sieht vor, dass jede datenhaltende Stelle (Data Holder) dem zuständigen HDAB zu jedem Datensatz eine Datenbeschreibung zukommen lassen muss. Diese Beschreibung muss einem festgelegten Standard entsprechen. Es ist vorgesehen, dass diese Beschreibungen der jeweiligen HDABs in einem Metadatenkatalog veröffentlicht und auf europäischer Ebene in einem europäischen Datenkatalog zusammengeführt werden. Dieser soll es den Nutzenden ermöglichen, alle dem EHDS angeschlossenen europäischen Datensätze zu finden [5, 10].

Die Joint Action TEHDAS hat in dieser Hinsicht mehrere wichtige Beiträge geleistet. Zum einen hat *Milestone 6.2* [14] existierende Metadatenstandards identifiziert und beschrieben. Das *Deliverable 6.2* hat Empfehlungen gegeben, welche Standards innerhalb des EHDS für „Data Discovery“ geeignet sind [15]. Zudem hat sich Arbeitspaket 7 damit auseinandergesetzt, welche Optionen für die technische Implementierung der nationalen Kataloge und des europäischen Katalogs, den die zentralen Dienste der EU betreuen, bestehen [16].

Basierend auf der Arbeit von TEHDAS und dem Verordnungsvorschlag ist es ein Ziel des HealthData@EU-Pilotprojekts, einen funktionierenden europäischen Metadatenkatalog für Gesundheitsdaten aufzubauen. Zunächst entwickelt das Projekt eine Erweiterung des DCAT-AP-Standards speziell für Gesundheitsdaten, die sogenannte „DCAT-AP health extension“. Bei DCAT-AP handelt es sich um eine Spezifikation für Metadatensätze, die an die spezifischen Anwendungsanforderungen von Datenportalen in Europa angepasst ist [10, 17]. Sobald diese Erweiterung verfügbar ist, wird sie in den Metadatenkatalogen der Datenplattformen, die an dem Projekt teilnehmen, implementiert. Die Europäische Kommission entwickelt als Teil der zentralen Dienste eine zentrale Verknüpfung, um die nationalen Metadatenkataloge in einem europäischen Katalog so zusammenzuführen, wie es in dem EHDS-Verordnungsentwurf vorgesehen ist [10].

#### Datengenehmigung/Datenzugang

Eine weitere Etappe innerhalb der EHDS User Journey ist die Datenzugangsanfrage. Die EHDS-Verordnung beschreibt in den Artikeln 45 und 46 die Bedingungen und Verfahren, um einen Datenzugang zu erhalten, sowie insbesondere die Rolle der HDABs.

TEHDAS hat sich in Arbeitspaket 5 vordergründig mit der allgemeinen Governance des zukünftigen EHDS beschäftigt. So hat *Deliverable 5.4* Governance-Optionen für den EHDS vorgelegt. In *Deliverable 5.3* wurden zudem verschiedene nationale Governance-Modelle beschrieben und verglichen. Zudem wurden Empfehlungen formuliert, wie grenzüberschreitende Projekte im Rahmen des EHDS ermöglicht und vereinfacht werden können [18].

Das HealthData@EU-Pilotprojekt baut ebenfalls für diese Etappe der EHDS User Journey eine Testversion auf. Basierend auf der Verordnungsvorlage und den Empfehlungen von TEHDAS arbeitet das Pilotprojekt innerhalb von Arbeitspaket 7 unter anderem an einer ersten europäischen Vorlage für die Datenzugangsanfrage anhand der EHDS-Verordnung sowie existierender Vorlagen und Erfahrungen auf nationaler Ebene [10].

### Interoperabilität und Datenqualität

Die Umsetzung eines grenzübergreifenden EHDS erfordert verbindliche europäische Anforderungen und gemeinsame Standards.

Die Sicherstellung der Datenqualität und gemeinsame Interoperabilitätsstandards sind von entscheidender Bedeutung und eine grundlegende Voraussetzung des EHDS zur Verbesserung der Nutzung von Gesundheitsdaten für sekundäre Zwecke. Dazu bedarf es europäischer Regelungen und Harmonisierung, um zu gewährleisten, dass Daten verlässlich und interoperabel in einem breiten Umfang für Forschung, Politikgestaltung und Regulierung verfügbar sind. Die semantische Interoperabilität ist essenziell, um Daten untereinander kommunizierbar zu machen.

Die Joint Action TEHDAS hat hierzu im Rahmen des Arbeitspakets 6 „Excellence in data quality“ wichtige Resultate und Lösungen für eine vertrauenswürdige sowie interoperable Sekundärdatennutzung erarbeitet. Die Resultate des Arbeitspakets enthalten Empfehlungen zur Sicherstellung der Datenqualität im EHDS, zur Anonymisierung von Daten und zum Umgang mit Datenungleichheiten für die Sekundärnutzung sowie Leitlinien für die Verwendung gemeinsamer semantischer Interoperabilitätsstandards. Somit soll sichergestellt werden, dass Gesundheitsdaten, die europaweit erhoben und für politische Entscheidungen, Regulierung und Forschung weiterverwendet werden, zuverlässig und kongruent für den jeweiligen Zweck geeignet sind. Das *Deliverable 6.2* „Recommendations to enhance interoperability within HealthData@EU“ hat hierzu Standards zur Interoperabilität identifiziert und beschrieben [14, 15].

Das HealthData@EU-Pilotprojekt knüpft an die Ergebnisse von TEHDAS an und befasst sich in Arbeitspaket 8 „Data interoperability, quality and protection“ mit der Interoperabilität, der Qualitätskontrolle und Qualitätssicherungsmechanismen, die seitens der im Netzwerk beteiligten Plattformen verschiedener Institutionen implementiert werden sollen. In enger Zusammenarbeit mit anderen Arbeitspaketen ist das primäre Ziel, unter Berücksichtigung der rechtlichen Grundlagen gültige Standards zu erproben [10].

#### Wegbereiter: IT-Infrastruktur

Ziel des TEHDAS Arbeitspakets 7 „Connecting the dots“ war es, Optionen für die technische Interoperabilität der Sekundärnutzung von Gesundheitsdaten im EHDS aufzuzeigen. Konkret wurde dies in Empfehlungen für die Architektur und Infrastruktur festgehalten. Leitlinien zu nationalen Datensätzen, Datenzugangsanträgen, Verwaltungssystemen für Datenanfragen und gesicherten Analyseräumen (Secure Processing Environments, SPEs) wurden erarbeitet. Des Weiteren wurden die Teilhabe und Teilnahme für zukünftige Nutzende am EHDS durch Stakeholder-Konsultationen gefördert. Es wurden interne Diskussionen über die Leitlinien der SPEs geführt. Außerdem wurden mittels externer Umfragen existierende SPE-ähnliche Infrastrukturen erfasst. Insgesamt ist der EHDS aufgrund seiner technischen Komplexität mit einem erheblichen Aufwand verbunden, der durch die ersten Grundlagenanalysen des Arbeitspakets eine erste Form erreicht hat [16].

Im HealthData@EU-Pilotprojekt koordinieren und leiten der HDH und das HDL am BfArM das Arbeitspaket 5 „IT-Infrastruktur“. Konkret bedeutet dies den Aufbau einer IT-Infrastruktur, welche die im Projekt involvierten Datenplattformen sowie die zentralen Dienste der Europäischen Kommission miteinander verbinden. Diese erste Netzwerkinfrastruktur des EHDS soll einen ersten Austausch von Informationen wie z. B. Metadaten ermöglichen und als technisches Rückgrat für weitere Komponenten fungieren. Ziel ist hierbei, zunächst die drei sogenannten Proof-of-Concept-Knoten (Frankreich, Dänemark und Finnland) und die zentralen Dienste über das aufgesetzte eDelivery-Netzwerk miteinander zu verbinden. Dieses Netzwerk zentralisiert die nationalen Datensatzkataloge und leitet die Anfragen zur Datengenehmigung von den zentralen Diensten zu den drei Knoten weiter. Im weiteren Verlauf des Projekts sollen sich weitere Institutionen sukzessive mit dem Netzwerk verbinden können. Hierzu kooperiert das Arbeitspaket 5 eng mit dem Arbeitspaket 6 „Metadaten-Standards“ und Arbeitspaket 7 „Beachtung von Vorschriften und Rechtsnormen“, die zuständig sind für die Erstellung von Spezifikationen für Datenanträge und -genehmigungen [10].

### Konsultation von Bürgerinnen und Bürgern/Stakeholdern

Von besonderer Bedeutung sind auch Konsultationen mit Bürgerinnen und Bürgern sowie Expertinnen und Experten, um Anregungen, Wünschen und Sorgen kontinuierlich zu begegnen und diese in den weiteren Aufbau des EHDS zu integrieren. Dazu wurde in TEHDAS ein eigenes Arbeitspaket, „Citizen Engagement“, unter der Leitung des HDH und des ungarischen National Healthcare Service Centers geschaffen. Das Ziel ist, ein besseres Verständnis für die Einstellung der Bürgerinnen und Bürger zur Nutzung ihrer Gesundheitsdaten zu erlangen. Außerdem wurden Möglichkeiten ermittelt, Bürgerinnen und Bürger über die Verwendung ihrer Gesundheitsdaten zu informieren und für die Vorteile, die die Sekundärnutzung von Daten bietet, zu sensibilisieren. Im Ergebnis sind Bürgerinnen und Bürger durchaus dazu bereit, ihre Gesundheitsdaten für eine öffentliche Nutzung zur Verfügung zu stellen. Gleichzeitig wurde deutlich, dass die Kontrolle über die eigenen Daten ein Schlüsselaspekt des Vertrauensaufbaus darstellt. Hinsichtlich des neuen Konzepts des Datenaltruismus konnte festgestellt werden, dass mehr Wissensvermittlung und praktische Vertiefung unabdingbar sind [19].

Das HealthData@EU-Pilotprojekt arbeitet einerseits mit den Stakeholdern zusammen und berücksichtigt andererseits im Rahmen der konkreten Umsetzung die Rechtsvorschriften wie die DSGVO [10].

## Fazit

Insgesamt eröffnen der EHDS und die damit verbundenen rechtlichen Voraussetzungen neue und richtungsweisende Möglichkeiten, die Gesundheitsversorgung der Bevölkerung und die wissenschaftlichen Erkenntnisse auf europäische Ebene unter Einhaltung der Datenschutzstandards zu steigern. Durch die Kooperation in Europa wird Gesundheit zu einem grenzübergreifenden Thema. Der EHDS schafft die Grundlage für die technische und semantische Interoperabilität innerhalb Europas, die zu neuen Chancen der medizinischen Forschung beitragen und folglich gesamtgesellschaftlichen Nutzen im Hinblick auf die Gesundheitsversorgung aller Bürgerinnen und Bürger der EU hat. Die initiierten Projekte und die daraus hervorgehenden Ergebnisse tragen alle dazu bei, die Vision eines EHDS schrittweise umzusetzen und die gesundheitsbezogenen Herausforderungen gemeinschaftlich zu bewältigen.
